# Parents’ Adverse and Positive Childhood Experiences and Offspring Involvement With the Criminal Legal System

**DOI:** 10.1001/jamanetworkopen.2023.39648

**Published:** 2023-10-25

**Authors:** Elizabeth S. Barnert, Lindsay M. Schlichte, Destiny G. Tolliver, Jaime La Charite, Christopher Biely, Rebecca Dudovitz, Kathryn Leifheit, Shirley Russ, Narayan Sastry, Cecile Yama, George M. Slavich, Adam Schickedanz

**Affiliations:** 1 Department of Pediatrics, David Geffen School of Medicine at UCLA, Los Angeles, California; 2 Duke University School of Medicine, Durham, North Carolina; 3 Department of Pediatrics, Boston University Chobanian and Avedisian School of Medicine, Boston, Massachusetts; 4 Department of General Internal Medicine at UCLA, Los Angeles, California; 5 Institute for Social Research, University of Michigan, Ann Arbor; 6 Department of Psychiatry and Biobehavioral Sciences, University of California, Los Angeles; 7 Department of Health Policy and Management, UCLA Fielding School of Public Health, University of California, Los Angeles

## Abstract

**Importance:**

Intergenerational cycles of adversity likely increase one’s risk of criminal legal system involvement, yet associations with potential contributors, such as parents’ adverse childhood experiences (ACEs) and positive childhood experiences (PCEs), have not been explored.

**Objective:**

To investigate the association of parents’ ACEs and PCEs with their adult children’s involvement in US legal systems, from arrest to conviction.

**Design, Setting, and Participants:**

The study team analyzed data from the Panel Study of Income Dynamics (PSID), a nationally representative cohort study of families in the US. PSID-2013 survey data were merged with the 2014 PSID Childhood Retrospective Circumstances Study (CRCS), collected May 2014 to January 2015, which asked adults aged 18 to 97 years to retrospectively report on their childhood experiences. Parents and their adult children were linked in the data set. Data were analyzed from October 2022 to September 2023.

**Main Outcomes and Measures:**

The child arrest outcome was regressed on parents’ ACE and PCE scores using logistic regression models. In addition, multinomial logistic regression models were used to assess the associations of parents’ ACE and PCE scores with the number of times their child was arrested and convicted.

**Results:**

Of 12 985 eligible individuals, 8072 completed the CRCS. Among CRCS participants, there were 1854 eligible parent-child dyads (ie, parents and their adult children) that formed the analytic sample. The mean (SD) age of offspring at the time of CRCS completion was 38.5 (10.9) years, and 1076 offspring (51.3%) were female. Having 4 or more parental ACEs was associated with 1.91-fold (95% CI, 1.14-3.22) higher adjusted odds of arrest before age 26 and 3.22-fold (95% CI, 1.62-6.40) higher adjusted odds of conviction before age 26 years, compared with children of parents without ACEs. These associations persisted after controlling for parental PCEs.

**Conclusions and Relevance:**

In this nationally representative study, children of parents with higher ACEs were at greater risk of arrest during adolescence and young adulthood, even after controlling for parents’ PCEs. Addressing and preventing childhood adversity through multigenerational life course approaches may help disrupt intergenerational pathways to the criminal legal system.

## Key Points

**Question:**

Are parents’ adverse childhood experiences (ACEs) and positive childhood experiences associated with their children’s involvement in criminal legal systems across the carceral continuum?

**Findings:**

This cohort study of 1854 adult children found that having 4 or more parental ACEs was associated with 1.91-fold higher adjusted odds of arrest and 3.22-fold higher adjusted odds of conviction before age 26 years, compared with children of parents without ACEs. Associations persisted after controlling for parental positive childhood experiences.

**Meaning:**

These findings suggest that higher parental ACEs are associated with greater odds of their children’s arrest and conviction in the US criminal legal system; addressing and preventing childhood adversity through multigenerational life course approaches may help disrupt intergenerational pathways to the criminal legal system.

## Teaser

This cohort study investigates the association of parents’ adverse and positive childhood experiences with their adult children’s involvement in US legal systems, from arrest to conviction.

## Introduction

Adverse childhood experiences (ACEs), defined as potentially traumatic stressors in childhood, such as abuse, neglect, and household challenges,^1^ are prevalent and associated with negative health outcomes throughout life.^2^ ACEs are associated with legal system contact, including arrests, incarceration, and longer time served.^3,4^ Emerging research on positive childhood experiences (PCEs) suggests that PCEs can modify or buffer associations between ACEs and youth criminal legal system involvement.^5,6,7,8^ ACEs have the potential to transmit across generations, whereby childrearing practices and social and biological circumstances are influenced by parents’ childhood trauma.^9,10^ However, a lack of cross-generational studies means there is little evidence as to whether parents’ ACEs and PCEs are associated with intergenerational risk of legal system contact for their offspring.

Few published studies have examined the impact of parental ACEs or PCEs on their children beyond infancy and early childhood, and child outcomes have not included legal system involvement. Efforts to decrease the risk of criminal legal system involvement may be enhanced by examining parents’ ACE and PCEs and their associations with their children’s outcomes throughout their life course.^10,11,12,13,14,15,16,17,18,19,20,21^ Such studies could inform the development of interventions that support lifelong and intergenerational trajectories of health and well-being.^22,23,24,25^ Considering parents’ exposures to ACEs and PCEs may help identify children most at risk in order to develop and implement programs and policies that can alter children’s trajectories away from entanglement in the criminal legal system.

To our knowledge, no prior studies have examined associations of parents’ ACEs with young people’s involvement in the criminal legal system using national data. We, therefore, examined the association of parental ACEs with their children’s involvement in the legal system across the carceral continuum from arrest to conviction, while also measuring how PCEs might mediate this association, in a national sample of US families.

## Methods

### Study Sample

The University of California, Los Angeles, institutional review board approved this study, which uses restricted data under contract from the University of Michigan’s Institute for Social Research. This study follows the Strengthening the Reporting of Observational Studies in Epidemiology (STROBE) reporting guideline for cohort studies. Our study sample comprised parent-child dyads of parents and their adult children participating in the Panel Study of Income Dynamics (PSID), a nationally representative US panel survey with genealogic design. Participants provided informed consent through the survey. The PSID is sponsored by the National Science Foundation and National Institutes of Health and collects data biennially from 10 000 families. Families were recruited at baseline and in a 1997 new immigrant refresher sample, as well as when adult children split off to form their own independent family units.^26^ We used PSID-2014 Childhood Retrospective Circumstances Study (CRCS)^26^ and linked parents and their adult children in the data set using family relationship identifiers from PSID’s Family Identification Mapping System. CRCS was collected via web and mail as a supplement to the biennial PSID survey, collected by telephone interview. CRCS asked adults (aged 18-97 years) to retrospectively report their childhood experiences, including ACEs and PCEs. Eligible respondents were English-speaking adults and their spouses or partners from the PSID-2013 interview. Families from PSID-2013 with a proxy respondent or who were interviewed in Spanish were deemed ineligible for PSID-CRCS (593 families).

All parent-child dyads with complete data on child arrests (primary outcome), at least 1 parental ACE score (primary variable), and a sample weight were included in the analytic sample (eFigure in Supplement 1). Individual and household-level covariates were drawn from the PSID-2013 and merged with the analytic sample.

### Measures

#### Outcome: Criminal Legal Involvement

The primary outcome was adult child arrest before age 26 years; this age range encompasses childhood through the transition age youth period (ie, older adolescence to young adulthood).^27^ For ease of interpretation, we use the term *child* to refer to the younger generation in the parent-child dyads, although criminal legal involvement may have occurred during their young adulthood.

Arrest and conviction history was reported by the adult child in CRCS. Participants were asked whether they had been arrested (primary outcome), number of arrests, age at first arrest, whether convicted, number of convictions, and age at first conviction. Consistent with prior studies, outcome variables were constructed to measure any adult child arrest, number of times arrested (never, once, or more than once), any conviction, and number of times convicted before age 26 years (never, once, or more than once).^23^

#### ACE Measures

Parents participating in CRCS reported conventional ACEs before age 18 years, including physical abuse, emotional abuse, sexual abuse or assault, emotional neglect, witnessing intimate partner violence at home, witnessing household substance use, having a parent with mental illness, any parental separation or divorce, and/or having a deceased or estranged parent. Parental arrest history was not included in the proxy ACE score construction. ACE counts were binned into 4 categories (0, 1, 2-3, and ≥4), consistent with prior research.^28,29^ For the main analysis, if both parents had ACE scores, the higher ACE score across both parents was used; if only 1 parent participated in CRCS, their ACE score was used (eAppendix 1 and eTable 1 in Supplement 1).

#### PCE Measures

CRCS included questionnaire items on respondents’ relationships with caregivers, friends, and neighbors, as well as experiences of their neighborhood and school environments. The CRCS items are similar to commonly used and psychometrically validated PCE measures.^30,31,32^ We constructed a proxy PCE measure from these items using the method from prior analyses performed by 2 of the coauthors (J.L. and A.S.). Our final PCE measure created a binned PCE score (0-1, 2-3, and 4-5 PCEs) (eAppendix 2 and eTable 2 in Supplement 1).

### Covariates

We selected covariates potentially associated with both parents’ ACEs and children’s criminal legal system involvement.^17^ These included adult child age, sex, race, Latino ethnicity, highest level of parental education, and highest level of parental income. PSID uses racial and ethnic category conventions used by the federal government based on standards established by the Federal Office of Management and Budget. We used the PSID race categories selections, which included African American, Asian/Pacific Islander, White, and other (specify). When appropriate, some of the other (specify) responses were coded into the provided response options. Survey respondents self-identified race and ethnicity, or it was reported by a proxy (spouse or partner). We included these reported categories as covariates to serve as markers for racism and discrimination that are associated with both exposure to childhood adversity and contact with the criminal legal system within and across generations.

### Statistical Analysis

Data were analyzed from October 2022 to September 2023. We examined sample characteristics with descriptive statistics. Sociodemographic characteristics were compared with arrest outcomes using the designed-based *F* test for categorical variables and the adjusted Wald *F* test for continuous variables. For our main analysis, we regressed the child arrest outcome on parents’ ACE and PCE scores using logistic regression models, adjusting for covariates. In addition, we used multinomial logistic regression models and calculated estimated probabilities to assess the associations of parents’ ACE and PCE scores with the number of times arrested and convicted outcomes variables, adjusting for covariates. Multinomial logistic regression allowed us to analyze our categorical outcome without relying on a proportional odds assumption. We used robust variance estimation to account for clustering within families. Analyses were performed in Stata statistical software version 17.0 (StataCorp), using the svy commands. Two-sided *P* < .05 was considered statistically significant. Analyses were weighted to address the complex survey sampling, nonresponse, and achieve population representation.

## Results

 Of 12 985 eligible individuals, 8072 completed CRCS between May 2014 and January 2015, for an unweighted response rate of 62.2% (weighted response rate 67.0%), which is similar to response rates for web-based supplements to other national panel studies.^26^ The 8072 CRCS participants included 2164 parent-child dyads. Of the analytic sample of 1854 parent-child dyads with complete data, 348 (18.3%) children experienced arrest before age 26 years, the mean (SD) offspring participant age was 38.5 (10.9) years, and 1076 offspring (51.3%) were female (Table). The experience of arrest by age 26 years differed by sex, affecting 227 male participants (27.8%) and 121 female participants (9.4%). Race, ethnicity, parental education, and parental income did not differ between those with and without arrest before age 26 years. Frequencies and weighted percentages of parental ACEs among adult children were 582 (31.6%) for physical abuse, 384 (19.7%) for emotional abuse, 49 (2.3%) for sexual abuse or assault, 193 (10.8%) for emotional neglect, 439 (21.7%) for witnessing intimate partner violence at home, 430 (22.5%) for witnessing household substance use, 449 (24%) for having a parent with mental illness, 432 (18.8%) for parental separation or divorce, and 127 (6.9%) for having a deceased or estranged parent. Among participants, 881 (47.5%) had only maternal ACEs data, 324 (17.5%) had only paternal ACEs data, and 649 (35.0%) had ACEs data from both parents. Adult children with more parental ACEs had higher prevalence of arrest by age 26 years. In unadjusted bivariate analyses, the numbers of adult children who had been arrested before age 26 years according to ACEs reported by their parents were as follows: 0 ACEs, 83 children (17.2%); 1 ACE, 101 children (17.5%); 2 to 3 ACES, 112 children (16.9%), and 4 or more ACEs, 52 children (27.8%) (Table). There was no significant difference between number of parental ACEs and likelihood of arrest. The numbers of adult children who were arrested by age 26 years according to parental PCEs were as follows: 0 or 1 PCE, 110 children (20.5%); 2 or 3 PCEs, 168 children (18.7%); and 4 or 5 PCEs, 70 children (14.9%). There was no significant difference between number of parental PCEs and likelihood of arrest (Table).

**Table.** Characteristics of Adult Children

Characteristic	Participants, No. (weighted %)			*P* value^a^
	Overall (N = 1854)	Never arrested before age 26 y (n = 1506)	Arrested before age 26 y (n = 348)	
Age, mean (SD), y	38.5 (10.9)	39.0 (10.9)	36.3 (10.4)	.002
Sex				
Male	778 (48.7)	551 (72.2)	227 (27.8)	<.001
Female	1076 (51.3)	955 (90.6)	121 (9.4)	
Race				
African American	483 (10.0)	369 (76.3)	114 (23.7)	.47
Asian or Pacific Islander	27 (1.9)	23 (88.5)	4 (11.5)	
White	1316 (87.4)	1088 (82.0)	228 (18.0)	
Other^b^	14 (0.8)	13 (82.0)	1 (18.0)	
Latino or Hispanic ethnicity	75 (4.4)	60 (80.4)	15 (19.6)	.85
Highest parental education				
Less than high school	192 (8.7)	154 (85.4)	38 (14.6)	.41
High school graduate or equivalent	445 (23.2)	357 (79.8)	88 (20.2)	
College, vocational school, or graduate school	1217 (68.1)	995 (81.8)	222 (18.2)	
Highest parental income, % of Federal Poverty Level				
<100	160 (5.5)	120 (73.1)	40 (26.9)	.38
100-199	276 (12.4)	220 (83.5)	56 (16.5)	
200-299	275 (15.9)	227 (83.2)	48 (16.8)	
300-400	216 (12.6)	177 (79.7)	39 (20.3)	
>400	927 (53.6)	762 (82.1)	165 (17.9)	
No. of parental adverse childhood experiences^c^				
0	502 (30.2)	419 (82.8)	83 (17.2)	.05
1	605 (32.4)	504 (82.5)	101 (17.5)	
2-3	550 (26.7)	438 (83.1)	112 (16.9)	
4 or more	197 (10.7)	145 (72.2)	52 (27.8)	
No. of parental positive childhood experiences^c^				
0-1	512 (27.9)	402 (79.5)	110 (20.5)	.24
2-3	924 (50.2)	756 (81.3)	168 (18.7)	
4-5	418 (21.9)	348 (85.1)	70 (14.9)	
Mother’s education				
Less than high school	224 (10.5)	181 (85.8)	43 (14.2)	.31
High school graduate or equivalent	516 (27.2)	419 (79.8)	97 (20.2)	
College, vocational school, or graduate school	1114 (62.2)	906 (81.8)	208 (18.2)	
Father’s education				
Less than high school	105 (9.6)	83 (77.6)	22 (22.4)	.35
High school graduate or equivalent	285 (24.0)	242 (85.1)	43 (14.9)	
College, vocational school, or graduate school	646 (66.5)	548 (84.2)	98 (15.8)	

^a^ We used an adjusted Wald *F* test to determine whether there were differences in means across being arrested before age 26 years for continuous variables, and we used a design-based *F* test to determine whether there were associations between being arrested before age 26 years and categorical variables.

^b^ The Panel Study of Income Dynamics survey asks participants to specify what they mean by other race.

^c^ Indicates highest number of mother or father adverse childhood experiences or positive childhood experiences, respectively.

### Parent ACEs, Parent PCEs, and Their Children’s Arrests

**Figure 1.**
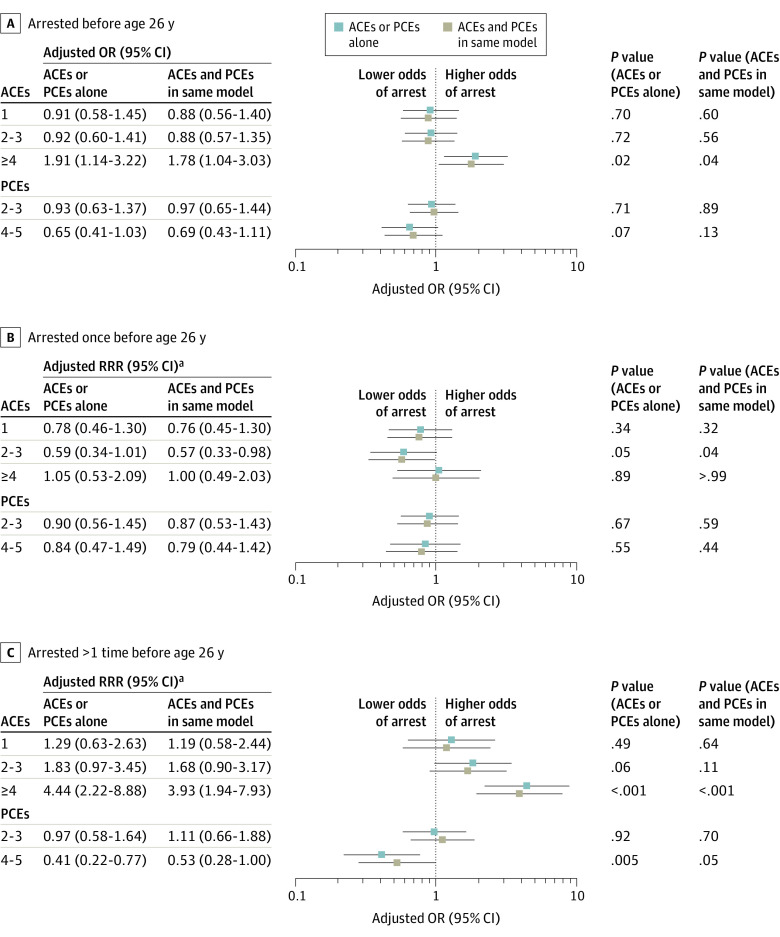
Adult Child Arrests Before Age 26 Years by Parental Adverse Childhood Experiences (ACEs) and Positive Childhood Experiences (PCEs) Forest plots show adjusted odds ratios (ORs) for all adult children arrested (A) and adjusted relative risk ratios (RRRs) for those arrested once (B) and those arrested more than once (C). Models were adjusted for adult child age, sex, race, Latino ethnicity, highest level of parental education, and highest level of parental income. ^a^RRR was calculated using coefficients derived from multinomial logistic regression, comparing each outcome with a base outcome of no arrest.

Compared with offspring with no parental ACEs, having 4 or more parental ACEs was associated with a higher odds of arrest before age 26 years, both before (adjusted odds ratio [aOR], 1.91; 95% CI, 1.14-3.22) and after (aOR, 1.78; 95% CI, 1.04-3.03) adjusting for PCEs and covariates (Figure 1; eTable 3 in Supplement 1). In absolute terms, children of parents with 4 or more ACEs had an estimated probability of being arrested by age 26 years of 28.0% (95% CI, 20.0%-35.0%) vs 18.0% (95% CI, 14.0%-23.0%) among children of parents with no ACEs (eTable 4 in Supplement 1).

Among offspring with arrest histories, the mean (SD) number of arrests per individual before age 26 years was 2.48 (5.80). Compared with adult children without parental ACEs, having 4 or more parental ACEs was associated with a higher risk of being arrested more than once vs no arrest, adjusting for PCEs and covariates (adjusted relative risk ratio [RRR], 3.93; 95% CI, 1.94-7.93) (Figure 1; eTable 4 in Supplement 1). Compared with adult children with 0 or 1 parental PCE, having 4 or 5 parental PCEs was associated with a lower risk of multiple arrests vs no arrest before controlling for ACEs (adjusted RRR, 0.41; 95% CI, 0.22-0.77). However, after incorporation of parental ACEs into the model, the association was no longer statistically significant (adjusted RRR, 0.53; 95% CI, 0.28-1.00) (Figure 1; eTable 3 in Supplement 1). eTable 4 in Supplement 1 presents estimated probabilities.

### Parent ACEs, Parent PCEs, and Their Children’s Convictions

**Figure 2.**
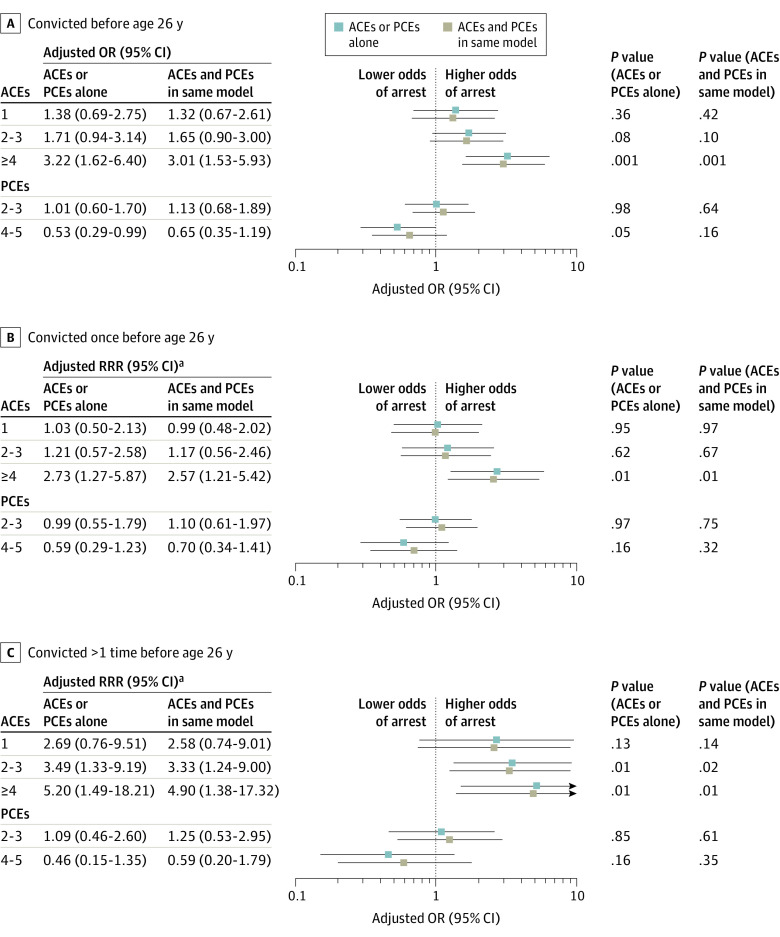
Adult Child Convictions Before Age 26 Years by Parental Adverse Childhood Experiences (ACEs) and Positive Childhood Experiences (PCEs) Forest plots show adjusted odds ratios (ORs) for all adult children convicted (A) and adjusted relative risk ratios (RRRs) for those convicted once (B) and those convicted more than once (C). Models were adjusted for adult child age, sex, race, Latino ethnicity, highest level of parental education, and highest level of parental income. ^a^RRR was calculated using coefficients derived from multinomial logistic regression, comparing each outcome with a base outcome of no conviction.

Compared with offspring without parental ACEs, having 4 or more parental ACEs was associated with a higher odds of conviction before (aOR, 3.22; 95% CI, 1.62-6.40) and after (aOR, 3.01; 95% CI, 1.53-5.93) controlling for parental PCEs (Figure 2; eTable 5 in Supplement 1). Having 4 to 5 parental PCEs was associated with a lower odds of being convicted compared with adult children with 0 or 1 parental PCE (aOR, 0.53; 95% CI, 0.29-0.99) before controlling for ACEs. However, this association was no longer significant in the model examining both parental ACEs and PCEs (aOR, 0.65; 95% CI, 0.35-1.19) (Figure 2; eTable 5 in Supplement 1). In absolute terms, children of parents with 4 or more ACEs had an estimated probability of being convicted of 14.0% (95% CI, 8.0%-20.0%) compared with 5.0% (95% CI, 3.0%-8.0%) among children of parents with 0 ACEs (eTable 6 in Supplement 1).

Compared with offspring without parental ACEs, having 4 or more parental ACEs was associated with a higher risk of 1 conviction (adjusted RRR, 2.57; 95% CI, 1.21-5.42) or multiple convictions (adjusted RRR, 4.90; 95% CI, 1.38-17.32) vs no convictions, adjusting for PCEs and covariates (Figure 2; eTable 5 in Supplement 1). The estimated probability was 9.0% (95% CI, 5.0%-13.0%) for 1 conviction and 5.0% (95% CI, 0.0%-10.0%) for multiple convictions (eTable 6 in Supplement 1).

In sensitivity analyses examining ACEs and PCEs as continuous rather than categorical variables, neither number of parental ACEs nor PCEs was significantly associated with adult child arrest by age 26 years. However, increasing parental ACE scores were associated with a higher adjusted odds of conviction before age 26 years after controlling for parental PCEs (aOR, 1.22; 95% CI, 1.06-1.39) (eTable 7 in Supplement 1).

## Discussion

Overall, this cohort study found an association between parents’ ACEs and their child’s criminal legal system involvement during the critical life course transition of adolescence and young adulthood, suggesting potential intergenerational sequelae of ACE exposure among people in the US. More than 1 in 6 survey participants experienced arrest before age 26 years, consistent with prior published rates in US samples.^33^ The odds of being arrested before the age of 26 years for children of parents with 4 or more ACEs was nearly double (1.78 times) that of children whose parents reported 0 or 1 ACE. Even after controlling for parental PCEs, the associations of parental ACEs with their adult child’s criminal legal system involvement persisted. The high prevalence of ACEs—and the health inequities and racial injustice exacerbated by the US juvenile and adult criminal legal systems—make this finding especially alarming.^23,34^

### Life Course Lens on ACEs, PCEs, and Youth Criminal Legal Involvement

Potential factors contributing to the observed association between parent ACEs and adult child’s arrest may include intergenerational transmission of trauma, shared familial risks (eg, genetic propensity for developmental delay, behavioral health challenges, or other factors associated with the development of criminalized behaviors), and structural disadvantage and oppression (eg, racism and social or economic disadvantage).^10,12,14,16,18,19,20,21,29^ For example, evidence suggests that parental mental health symptoms can be a consequence of parents’ ACEs, and parental trauma may influence parenting behaviors.^10,24^ Parental mental illness symptoms may serve as a powerful link in the parent-to-child ACEs continuity, and mental illness in children greatly increases the risk of criminal legal involvement.^35^

Parents with a history of ACEs who also experience PCEs display more positive long-term functioning^24^; however, our study may have failed to show a protective effect of parent PCEs because of limited power, as suggested by nonsignificant but decreasing odds of arrests and convictions with increasing parental PCEs. It is possible that parental ACEs are especially impactful on pathways to offspring criminal legal system involvement in ways that are relatively unaffected by parent PCEs,^36^ suggesting a need for a different approach to ACEs mitigation.

### Policy Implications

Our findings suggest that there is a crucial need for prevention of parent ACE exposure in the first place, as well as efforts to mitigate the impact of ACEs on families before they have downstream impacts on the next generation. The magnitude of associations between parent ACEs and young people’s arrest and conviction outcomes provide evidence that can be motivating to policymakers and may help shape paradigms away from blaming young people, instead stimulating investments in the supports they and their families need to thrive. Interventions such as nurse home visiting programs and the Perry preschool project have demonstrated strong effects in improving young people’s criminal legal outcomes.^37,38^ On the basis of prior research, we expected a possible mitigating effect of PCEs; however, our findings suggested that PCEs were overall not substantial enough to overcome the deleterious impact of ACEs. Although unexpected, this finding is consistent with those of prior studies,^24,39,40,41^ which have shown an inconsistent association of PCEs with outcome measures once accounting for ACEs, ranging from no effect of PCEs to a protective effect at certain levels of ACE exposure or within certain subgroups. Furthermore, youth incarceration is known to have substantial damaging effects on a person’s health and social success well into adulthood and is highly overused in the US, especially among communities of color and for people with multiple marginalized intersectional identities.^23^ To achieve health equity, prevention of ACEs, coupled with dismantling of a carceral system that is racially biased and counterproductive, is an important focus for policymakers. Parental adversity begets later childhood adversity, and it is vital that every child be given the best chance at a healthy start.^21^ Early ACE identification can help detect risk, and a timely and effective response to a child’s identified ACE is paramount, as is a societal reorientation toward health equity and valuing the well-being of all children, including through poverty prevention measures such as the Supplemental Nutrition Assistance Program and elimination of the school-to-prison pipeline. Furthermore, ACEs screening and remediation that involves not only children but also considers parental history of childhood adversity may be impactful. Finally, our findings suggest that for young people with exposure to the juvenile or adult criminal legal systems, multigenerational, family-centered interventions may be warranted for ensuring optimal health trajectories.

### Limitations

This study has limitations that should be mentioned. Collection of ACEs and PCEs retrospectively is standard in the field, and prior studies^9,28,29^ have demonstrated that prospective and retrospective ACEs measures are largely concordant. Criminal legal involvement may be underreported because of social desirability bias and differential attrition of individuals with arrest histories compared with those without. In addition, our measures, like other ACEs and PCEs measures, were constructed and compiled from survey measures and were not psychometrically tested before being fielded. It was unclear whether we were underpowered to detect a significant association between PCEs and arrest, because the aORs for the higher PCE scores seemed meaningful but the 95% CIs were large. Although information on parent legal involvement was not included, our research augments the established link between parent incarceration and child incarceration by exploring the broader context of family-related factors.^11,13,14,15,16^ However, unmeasured confounding is always a possibility in observational studies, although we have attempted to account for likely individual-level and family-level confounders. Furthermore, our analyses did not identify potential mechanisms underlying the observed relationships, such as experiences of racism, and the relative influence of factors such as specific ACE type (vs ACE load), maternal vs paternal ACEs, and timing of ACE and PCE exposure in impacting a young person’s trajectory toward arrest remain unclear, suggesting ripe opportunities for further study.

## Conclusions

Adult children of parents with higher ACE exposure may be at greater risk of arrest and subsequent criminal legal exposure during adolescence and young adulthood, even after accounting for parental PCEs. Our study suggests that disrupting intergenerational transmission of risk from parental ACE exposure may help prevent life course pathways to the criminal legal system in the next generation. Doing so represents a crucial, presently underaddressed opportunity to advance health equity and improve the lives of children and their families for generations to come.

## Notes

Supplement 1. **eFigure.** Sample Inclusion Flowchart **eAppendix 1.** Variable Construction for ACE and PCE Measures **eTable 1.** Classification and Variable Construction for Adverse Childhood Experiences (ACEs) Using the Panel Study of Income Dynamics **eAppendix 2.** Construction of the Positive Childhood Experience (PCE) Measure **eTable 2.** Classification and Variable Construction for Positive Childhood Experiences (PCEs) Using the Panel Study of Income Dynamics **eTable 3.** Adult Child Arrests Before Age 26 by Parent ACEs and PCEs **eTable 4.** Adult Child Arrests Before Age 26 by Parent ACEs and PCEs With Predicted Probabilities **eTable 5.** Adult Child Convictions Before Age 26 by Parent ACEs and PCEs **eTable 6.** Adult Child Convictions Before Age 26 by Parent ACEs and PCEs With Predicted Probabilities **eTable 7.** Adult Child Arrests and Convictions Before Age 26 by Parent ACEs and PCEs (Continuous ACE and PCE Score)

Supplement 2.
Data Sharing Statement
