# Supplementary material for: Parents’ Adverse and Positive Childhood Experiences and Offspring Involvement With the Criminal Legal System
Source: JAMA Netw Open. 2023 Oct 25;6(10):e2339648. doi: 10.1001/jamanetworkopen.2023.39648 (PMC10600584; doi:10.1001/jamanetworkopen.2023.39648)
Supplement: Supplement 1. — eFigure. Sample Inclusion Flowchart eAppendix 1. Variable Construction for ACE and PCE Measures eTable 1. Classification and Variable Construction for Adverse Childhood Experiences (ACEs) Using the Panel Study of Income Dynamics eAppendix 2. Construction of the Positive Childhood Experience (PCE) Measure eTable 2. Classification and Variable Construction for Positive Childhood Experiences (PCEs) Using the Panel Study of Income Dynamics eTable 3. Adult Child Arrests Before Age 26 by Parent ACEs and PCEs eTable 4. Adult Child Arrests Before Age 26 by Parent ACEs and PCEs With Predicted Probabilities eTable 5. Adult Child Convictions Before Age 26 by Parent ACEs and PCEs eTable 6. Adult Child Convictions Before Age 26 by Parent ACEs and PCEs With Predicted Probabilities eTable 7. Adult Child Arrests and Convictions Before Age 26 by Parent ACEs and PCEs (Continuous ACE and PCE Score) [file jamanetwopen-e2339648-s001.pdf]

Supplemental Online Content

Barnert ES, Schlichte LM, Tolliver DG, et al. Parents ’ adverse and positive childhood experiences and offspring involvement with the criminal legal system. *JAMA Netw Open*. 2023;6(10):e2339648. doi:10.1001/jamanetworkopen.2023.39648

eFigure. Sample Inclusion Flowchart
eAppendix 1. Variable Construction for ACE and PCE Measures
eTable 1. Classification and Variable Construction for Adverse Childhood Experiences (ACEs) Using the Panel Study of Income Dynamics
eAppendix 2. Construction of the Positive Childhood Experience (PCE) Measure
eTable 2. Classification and Variable Construction for Positive Childhood Experiences (PCEs) Using the Panel Study of Income Dynamics
eTable 3. Adult Child Arrests Before Age 26 by Parent ACEs and PCEs
eTable 4. Adult Child Arrests Before Age 26 by Parent ACEs and PCEs With Predicted Probabilities
eTable 5. Adult Child Convictions Before Age 26 by Parent ACEs and PCEs
eTable 6. Adult Child Convictions Before Age 26 by Parent ACEs and PCEs With Predicted Probabilities
eTable 7. Adult Child Arrests and Convictions Before Age 26 by Parent ACEs and PCEs (Continuous ACE and PCE Score)

This supplemental material has been provided by the authors to give readers additional information about their work.

© 2023 Barnert ES et al. *JAMA Network Open.*

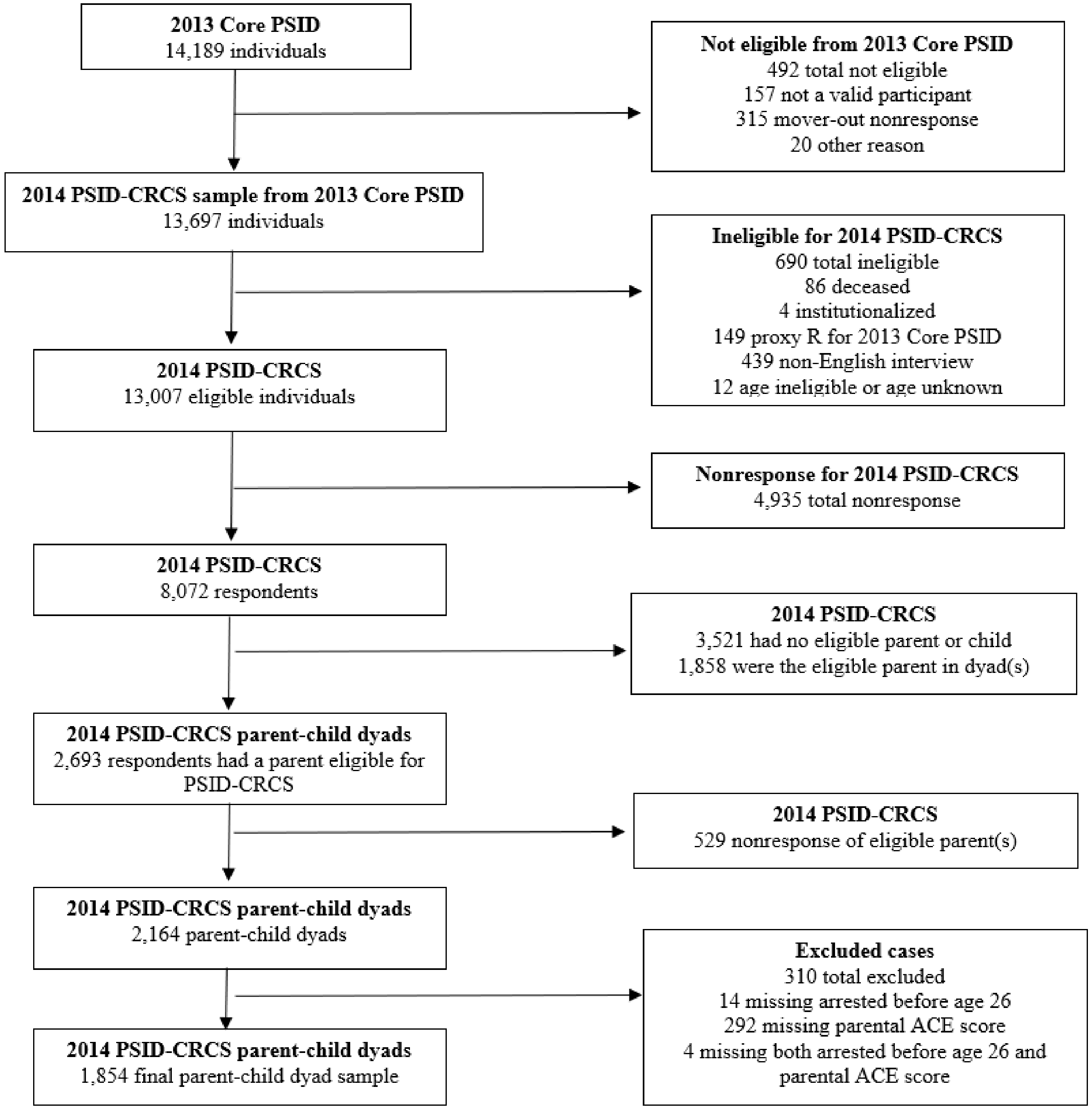

eFigure: Sample Inclusion Flowchart

© 2023 Barnert ES et al. *JAMA Network Open.*

eAppendix 1. Variable Construction for ACE and PCE Measures

Construction of the Adverse Childhood Experience (ACE) Measure

We matched the CRCS items to other existing validated ACE scales. We grouped similar CRCS items into conventional ACE domains based on the prior literature. We created eight binary variables to indicate whether an individual was exposed to a given ACE for each of the eight ACE domains. When there were multiple CRCS items within a given ACE domain, then a reported adverse experience for at least one of the items within the domain was defined as the respondent being exposed to the given ACE. Appendix B, Table 1 shows the CRCS items that were included for each of the ACE domains. We bolded the response options for each question that contributed to an affirmative ACE count for that ACE domain. For example, an individual that responded with a bolded answer to at least one of the following questions would be classified as experiencing the "Domestic Violence" ACE as a child.

1. Before you were age 17, how often did your mother and father push, grab, or shove each other? [**Often, Sometimes, Not Very Often**, Never, N/A]
2. Before you were age 17, how often did your mother and father throw something at each other? [**Often, Sometimes, Not Very Often**, Never, N/A]
3. Before you were age 17, how often did your mother and father slap or hit each other? [**Often, Sometimes, Not Very Often**, Never, N/A]
4. Before you were age 17, how often did your mother and father physically harm each other in any other way? [**Often, Sometimes, Not Very Often**, Never, N/A]

We summed across the binary to create a count score from 0 to 8. We then binned the scores into grouped categories: 0, 1, 2-3, and ≥4 ACEs, consistent with prior research. For the main analysis, the parent ACE predictor variable was specified as the higher of either parent's ACE count category, allowing for the inclusion of children with only one parent who participated in CRCS.

© 2023 Barnert ES et al. *JAMA Network Open.*

eTable 1: Classification and Variable Construction for Adverse Childhood Experiences (ACEs) Using the Panel Study of Income Dynamics

ACE Domain	Number of PSID Items	PSID Items
Neglect	3 total (2 for mother, 1 for father)	1. How much love and affection did your mother (including stepmother or the woman who raised you) give you? [**A lot, Some, A little**, None at all, N/A]<br>2. How much effort did you mother put into watching over you and making sure you had a good upbringing? [**A lot, Some, A little,** None at all, N/A]<br>3. Before you were age 17, how much love and affection did your father (including stepfather or the man who raised you) give you? [**A lot, Some, A little**, None at all, N/A]
Physical Abuse	6 (3 for mother, 3 for father)	1. Before you were age 17, how often did your mother [**Often, Sometimes, Not very often**, Never, N/A]<br>1b. Throw something at you?<br>1c. Slap or hit you?<br>1d. Physical harm in any other way?<br><br>*Repeat all for father*
Sexual Abuse	1	What was the crime that was committed against you? [if Assault (battery, rape aggravated assault, attempted manslaughter) was selected]
Emotional Abuse	2 (1 for mother, 1 for father)	1. Before you were age 17, how much tension did you have in your relationship with your mother (including stepmother or the woman who raised you? [**A lot, Some, A little**, None at all, N/A]<br><br>*Repeat identical question for father*
Parent Mental Health	4 (2 for mother, 2 for father)	1. Before you were age 17, did your mother ever have anxiety attacks where all of a sudden, she felt frightened, anxious or panicky? [**Y**/N]<br><br>2. Before you were age 17, did your mother ever have periods lasting 2 weeks or more where she was sad or depressed most of the time? [**Y**/N]<br><br>*Repeat both for father*
Parent Substance Use Disorder	10	1. Before you were age 17, did your mother have a problem with alcohol or drugs? [**Y**/N]<br><br>*Repeat identical question for father*
Parent Divorce or Separation	1	1. Thinking about your mother and father, did they separate or divorce during your childhood, that is, before you were 17? [**Y**/N]
Domestic Violence	4	1. Before you were age 17, how often did your mother and father push, grab, or shove each other? [**Often, Sometimes, Not very often**, Never, My parents were never together, N/A]<br><br>2. Before you were age 17, how often did your mother and father<br>2a. Throw something at each other<br>2b. Slap or hit each other?<br>2c. Physically harm each other in any other way?
Parent Incarcerated or Criminal Activity	0	0 CRCS items were available to populate this ACE domain.

ACE=Adverse Childhood Experience, PSID= Panel Study of Income Dynamics

© 2023 Barnert ES et al. *JAMA Network Open.*

eAppendix 2. Construction of the Positive Childhood Experience (PCE) Measure

We constructed the PCE measure for this study by matching the CRCS items with items in validated existing PCE scales to develop a PCE measure using the CRCS (Bethell et al, 2019; Guo et al, 2022; Narayan et al, 2018). In the CRCS, respondents were asked about their comfort with a group of friends and their safety and happiness at school. Second, they were asked to rate their childhood neighborhood in terms of its safety and whether neighbors were close knit and helped each other. Lastly, considering each parent separately (phrased in the survey as "the woman/man who raised you"), participants were asked to rate their relationship quality (e.g., communication, understanding, ability to confide in, closeness, overall assessment). If the respondent was not raised by a woman or man, then the respective item was coded to zero. We were unable to identify who was a child of a same sex couple for this study. See Table 2 for exact item language.

Based on comparisons of available, relevant items in CRCS to validated existing PCE scales, we grouped the relevant CRCS items into 5 domains. The five domains included items related to: (1) healthy school climate and supportive peer relationships, (2) neighborhood safety, (3) neighborhood support, (4) nurturing paternal relationship, and (5) nurturing maternal relationship. Responses to the available CRCS items within each domain were then averaged on the original CRCS item scale from 0 to 4 to create five domain measure averages. Similar to other studies, we dichotomized the PCE domain average measures at the 75th percentile to create five dichotomous variables that designated higher exposure to PCEs within each domain. Lastly, PCE scores were binned into grouped scores (0-1, 2-3, and 4-5), similar to ACE data. The table below displays the PSID CRCS items within each PCE domain.

eTable 2. Classification and Variable Construction for Positive Childhood Experiences (PCEs) Using the Panel Study of Income Dynamics

PCE Domain	Number of PSID Items	PSID Items
1) Peer Support and Healthy School Climate	6 questions (3 for age 6-12, 3 for age 13-16)	When you were between age 6 and 12<br>1. How often did you have a group of friends that you felt comfortable spending time with? [Often, Sometimes, Not very often, Never]<br>2. How often did you feel happy at school? [A lot, Some, A Little, Not at all]<br>3. How often did you feel worried about your physical safety at school? [A lot, Some, A Little, Not at all]<br><br>The same questions were asked for age 13 to 16.
2) Neighborhood Safety	6 questions (3 for age 6-12, 3 for age 13-16)	Think back to the neighborhood in which you lived the longest when you were between age 6 and 12. How true is each of the following statements about this neighborhood:<br>1. It was safe being out alone in my neighborhood at night. [very true, Somewhat true, Not very true, Not true at all]<br>2. My neighborhood was safe for children during the daytime. [Very true, Somewhat true, Not very true, Not true at all]<br>3. My neighborhood was safe for children during the nighttime. [Very true, Somewhat true, Not very true, Not true at all]<br><br>The same questions were asked for ages 13 to 16.
3) Neighborhood Support	4 questions (2 for age 6-12, 2 for age 13-16)	Think back to the neighborhood in which you lived the longest when you were between age 6 and 12. How true is each of the following statements about this neighborhood:<br>1. My neighbors were willing to help each other out. [Very true, Somewhat true, Not very true, Not true at all]<br>2. My neighborhood was close-knit. [Very true, Somewhat true, Not very true, Not true at all]<br><br>The same questions were asked for age 13 to 16.
4) Nurturing Maternal and 5) Paternal Relationship	5 questions for maternal, 5 questions for paternal	Before you were age 17,<br>1. How would you rate the communication between you and (your mother/your stepmother/the woman who raised you)? [Excellent, Very Good, Good, Fair, Poor]<br>2. How much could you confide in her about things that were bothering you? [A lot, Some, A Little, Not at all]<br>3. How much did (your mother/your stepmother/the woman who raised you) understand your problems and worries? [A lot, Some, A Little, Not at all. Before you were age 17: 4. How emotionally close were you with (your mother/your stepmother/the woman who raised you)? [Very, Somewhat, Not very, Not at all]<br>5. How would you rate your relationship with (your mother/your stepmother/the woman who raised you)? [Excellent, Very Good, Good, Fair, Poor]<br><br>The same questions were asked about "your father/your stepfather/the man who raised you."

[a]Based on prior studies, we summed across the five indicator variables for each of the five PCE domains to create a PCE count score from 0-5. We then grouped the PCE count score into three categories (0-1, 2-3, and 4-5 PCEs), as per prior studies (e.g., Guo et al, 2022).
PCE=Positive Childhood Experiences, PSID= Panel Study of Income Dynamics

© 2023 Barnert ES et al. *JAMA Network Open.*

eTable 3. Adult Child Arrests Before Age 26 by Parent ACEs and PCEs

Outcome: Arrested before Age 26						
	Adjusted[a] OR (95% CI) (*n*=1,840)	*p*-value	Adjusted[a] OR (95% CI) (*n*=1,840)	*p*-value	Adjusted[a] OR (95% CI) (*n*=1,840)	*p*-value
Number of Parental ACEs[b]						
0	reference				Reference	
1	0.91 (0.58, 1.45)	.70			0.88 (0.56, 1.40)	.60
2-3	0.92 (0.60, 1.41)	.72			0.88 (0.57, 1.35)	.56
4 or more	1.91 (1.14, 3.22)	.02			1.78 (1.04, 3.03)	.04
Number of Parental PCEs[b]						
0-1			reference		reference	
2-3			0.93 (0.63, 1.37)	.71	0.97 (0.65, 1.44)	.89
4-5			0.65 (0.41, 1.03)	.07	0.69 (0.43, 1.11)	.13
**Outcome: Number of Times Arrested before Age 26**						
	Adjusted[a] RRR[c] (95% CI) (*n*=1,840)	*p*-value	Adjusted[a] RRR[c] (95% CI) (*n*=1,840)	*p*-value	Adjusted[a] RRR[c] (95% CI) (*n*=1,840)	*p*-value
**Arrested Once before Age 26 (base outcome=no arrest)**						
Number of Parental ACEs[b]						
0	reference				reference	
1	0.78 (0.46, 1.30)	.34			0.76 (0.45, 1.30)	.32
2-3	0.59 (0.34, 1.01)	.05			0.57 (0.33, 0.98)	.04
4 or more	1.05 (0.53, 2.09)	.89			1.00 (0.49, 2.03)	>.99
Number of Parental PCEs[b]						
0-1			reference		reference	
2-3			0.90 (0.56, 1.45)	.67	0.87 (0.53, 1.43)	.59
4-5			0.84 (0.47, 1.49)	.55	0.79 (0.44, 1.42)	.44
**Arrested More than Once before Age 26 (base outcome=no arrest)**						
Number of Parental ACEs[b]						
0	reference				reference	

© 2023 Barnert ES et al. *JAMA Network Open.*

1	1.29 (0.63, 2.63)	.49			1.19 (0.58, 2.44)	.64
2-3	1.83 (0.97, 3.45)	.06			1.68 (0.90, 3.17)	.11
4 or more	4.44 (2.22, 8.88)	<.001			3.93 (1.94, 7.93)	<.001
Number of Parental PCEs[b]						
0-1			reference		reference	
2-3			0.97 (0.58, 1.64)	.92	1.11 (0.66, 1.88)	.70
4-5			0.41 (0.22, 0.77)	.005	0.53 (0.28, 1.00)	.05

[a]Model was adjusted for adult child age, sex, race, Latino ethnicity, highest level of parental education, and highest level of parental income.
[b]Indicates highest number of mother or father ACEs or PCEs, respectively.
[c] RRR was calculated using coefficients derived from multinomial logistic regression, comparing each outcome to a base outcome of no arrest.
ACE=adverse childhood experiences. PCE=positive childhood experiences. RRR=relative risk ratio. OR=odds ratio.

© 2023 Barnert ES et al. *JAMA Network Open.*

eTable 4: Adult Child Arrests Before Age 26 by Parent ACEs and PCEs With Predicted Probabilities

Outcome: Arrested before Age 26		
	Adjusted[a] OR (95% CI)	Predicted Probability[b] (95% CI)
Number of Parental ACEs[c]		
0	reference	0.18 (0.14, 0.23)
1	0.88 (0.56, 1.40)	0.17 (0.13, 0.21)
2-3	0.88 (0.57, 1.35)	0.17 (0.13, 0.21)
4 or more	1.78 (1.04, 3.03)*	0.28 (0.20, 0.35)
Number of Parental PCEs[c]		
0-1	reference	0.20 (0.15, 0.24)
2-3	0.97 (0.65, 1.44)	0.19 (0.16, 0.23)
4-5	0.69 (0.43, 1.11)	0.15 (0.11, 0.19)
**Outcome: Number of Times Arrested before Age 26**		
	Adjusted[a] RRR[d] (95% CI)	Predicted Probability[b] (95% CI)
**Once (base outcome=no arrest)**		
Number of Parental ACEs[c]		
0	reference	0.13 (0.09, 0.17)
1	0.76 (0.45, 1.30)	0.10 (0.07, 0.13)
2-3	0.57 (0.33, 0.98)*	0.08 (0.05, 0.10)
4 or more	1.00 (0.49, 2.03)	0.11 (0.05, 0.16)
Number of Parental PCEs[c]		
0-1	reference	0.11 (0.08, 0.15)
2-3	0.87 (0.53, 1.43)	0.10 (0.07, 0.13)
4-5	0.79 (0.44, 1.42)	0.10 (0.06, 0.14)
**More than once (base outcome=no arrest)**		
Number of Parental ACEs[c]		
0	reference	0.05 (0.03, 0.08)
1	1.19 (0.58, 2.44)	0.07 (0.04, 0.10)
2-3	1.68 (0.90, 3.17)	0.09 (0.06, 0.12)
4 or more	3.93 (1.94, 7.93)***	0.17 (0.11, 0.23)
Number of Parental PCEs[c]		
0-1	reference	0.08 (0.06, 0.11)
2-3	1.11 (0.66, 1.88)	0.09 (0.07, 0.12)
4-5	0.53 (0.28, 1.00)	0.05 (0.03, 0.07)

*p-value < .05; **p-value < .01; ***p-value < .001

[a]Model was adjusted for adult child age, sex, race, Latino ethnicity, highest level of parental education, highest level of parental income.

[b]We generated predicted probabilities by setting the other variables to their observed values.

[c]Indicates highest number of mother or father ACEs or PCEs, respectively.

© 2023 Barnert ES et al. *JAMA Network Open.*

[d]RRR was calculated using coefficients derived from multinomial logistic regression, comparing each outcome to a base outcome of no arrest.
ACE=adverse childhood experiences. PCE=positive childhood experiences. RRR=relative risk ratio. OR=odds ratio.

© 2023 Barnert ES et al. *JAMA Network Open.*

eTable 5: Adult Child Convictions Before Age 26 by Parent ACEs and PCEs

Outcome: Convicted before Age 26						
	Adjusted[a] OR (95% CI) (*n*=1,824)	*p*-value	Adjusted[a] OR (95% CI) (*n*=1,824)	*p*-value	Adjusted[a] OR (95% CI) (*n*=1,824)	*p*-value
Number of Parental ACEs[b]						
0	reference				reference	
1	1.38 (0.69, 2.75)	.36			1.32 (0.67, 2.61)	.42
2-3	1.71 (0.94, 3.14)	.08			1.65 (0.90, 3.00)	.10
4 or more	3.22 (1.62, 6.40)	.001			3.01 (1.53, 5.93)	.001
Number of Parental PCEs[b]						
0-1			reference		reference	
2-3			1.01 (0.60, 1.70)	.98	1.13 (0.68, 1.89)	.64
4-5			0.53 (0.29, 0.99)	.05	0.65 (0.35, 1.19)	.16
**Outcome: Number of Times Convicted before Age 26**						
	Adjusted[a] RRR[c] (95% CI) (*n*=1,837)	*p*-value	Adjusted[a] RRR[c] (95% CI) (*n*=1,837)	*p*-value	Adjusted[a] RRR[c] (95% CI) (*n*=1,837)	*p*-value
**Convicted Once before Age 26 (base outcome=no conviction)**						
Number of Parental ACEs[b]						
0	reference				reference	
1	1.03 (0.50, 2.13)	.95			0.99 (0.48, 2.02)	.97
2-3	1.21 (0.57, 2.58)	.62			1.17 (0.56, 2.46)	.67
4 or more	2.73 (1.27, 5.87)	.01			2.57 (1.21, 5.42)	.01
Number of Parental PCEs[b]						
0-1			reference		reference	
2-3			0.99 (0.55, 1.79)	.97	1.10 (0.61, 1.97)	.75
4-5			0.59 (0.29, 1.23)	.16	0.70 (0.34, 1.41)	.32
**Convicted More than Once before Age 26 (base outcome=no conviction)**						
Number of Parental ACEs[b]						
0	reference				reference	
1	2.69 (0.76, 9.51)	.13			2.58 (0.74, 9.01)	.14
2-3	3.49 (1.33, 9.19)	.01			3.33 (1.24, 9.00)	.02
4 or more	5.20 (1.49, 18.21)	.01			4.90 (1.38, 17.32)	.01
Number of Parental PCEs[b]						
0-1			reference		reference	
2-3			1.09 (0.46, 2.60)	.85	1.25 (0.53, 2.95)	.61

© 2023 Barnert ES et al. *JAMA Network Open.*

4-5			0.46 (0.15, 1.35)	.16	0.59 (0.20, 1.79)	.35

[a]Model was adjusted for adult child age, sex, race, Latino ethnicity, highest level of parental education, and highest level of parental income.
[b]Indicates highest number of mother or father ACEs or PCEs, respectively.
[c] RRR was calculated using coefficients derived from multinomial logistic regression, comparing each outcome to a base outcome of no conviction.
ACE=adverse childhood experiences. PCE=positive childhood experiences. RRR=relative risk ratio. OR=odds ratio.

© 2023 Barnert ES et al. *JAMA Network Open.*

eTable 6: Adult Child Convictions Before Age 26 by Parent ACEs and PCEs With Predicted Probabilities

Outcome: Convicted before Age 26		
	Adjusted[a] OR (95% CI)	Predicted Probability[b] (95% CI)
Number of Parental ACEs[c]		
0	reference	0.05 (0.03, 0.08)
1	1.32 (0.67, 2.61)	0.07 (0.04, 0.10)
2-3	1.65 (0.90, 3.00)	0.09 (0.06, 0.11)
4 or more	3.01 (1.53, 5.93)**	0.14 (0.08, 0.20)
Number of Parental PCEs[c]		
0-1	reference	0.08 (0.05, 0.11)
2-3	1.13 (0.68, 1.89)	0.09 (0.06, 0.11)
4-5	0.65 (0.35, 1.19)	0.05 (0.03, 0.08)
**Outcome: Number of Times Convicted before Age 26**		
	Adjusted[a] RRR[d] (95% CI)	Predicted Probability[b] (95% CI)
**Once (base outcome=no conviction)**		
Number of Parental ACEs[c]		
0	reference	0.04 (0.02, 0.06)
1	0.99 (0.48, 2.02)	0.04 (0.02, 0.06)
2-3	1.17 (0.56, 2.46)	0.05 (0.02, 0.07)
4 or more	2.57 (1.21, 5.42)*	0.09 (0.05, 0.13)
Number of Parental PCEs[c]		
0-1	reference	0.05 (0.03, 0.07)
2-3	1.10 (0.61, 1.97)	0.05 (0.03, 0.07)
4-5	0.70 (0.34, 1.41)	0.04 (0.02, 0.05)
**More than once (base outcome=no conviction)**		
Number of Parental ACEs[c]		
0	reference	0.01 (0.00, 0.02)
1	2.58 (0.74, 9.01)	0.03 (0.01, 0.06)
2-3	3.33 (1.24, 9.00)*	0.04 (0.02, 0.06)
4 or more	4.90 (1.38, 17.32)*	0.05 (0.00, 0.10)
Number of Parental PCEs[c]		
0-1	reference	0.03 (0.01, 0.05)
2-3	1.25 (0.53, 2.95)	0.03 (0.02, 0.05)
4-5	0.59 (0.20, 1.79)	0.02 (0.00, 0.03)

*p-value < .05; **p-value < .01; ***p-value < .001

[a]Model was adjusted for adult child age, sex, race, Latino ethnicity, highest level of parental education, highest level of parental income.

[b]We generated predicted probabilities by setting the other variables to their observed values.

[c]Indicates highest number of mother or father ACEs or PCEs, respectively.

[d]RRR was calculated using coefficients derived from multinomial logistic regression, comparing each outcome to a base outcome of no conviction.

ACE=adverse childhood experiences. PCE=positive childhood experiences. RRR=relative risk ratio. OR=odds ratio.

© 2023 Barnert ES et al. *JAMA Network Open.*

eTable 7. Adult Child Arrests and Convictions Before Age 26 by Parent ACEs and PCEs (Continuous ACE and PCE Score)

**Outcome: Arrested before the Age of 26**			
	Adjusted[a] OR (95% CI) (*n*=1,840)	Adjusted[a] OR (95% CI) (*n*=1,840)	Adjusted[a] OR (95% CI) (*n*=1,840)
Number of Parental ACEs(Higher of Either Parent's Score)	1.11 (1.00, 1.24)		1.09 (0.97, 1.22)
Number of Parental PCEs(Higher of Either Parent's Score)		0.90 (0.80, 1.01)	0.92 (0.82, 1.03)
**Outcome: Convicted before Age 26**			
	Adjusted[a] OR (95% CI) (*n*=1,824)	Adjusted[a] OR (95% CI) (*n*=1,824)	Adjusted[a] OR (95% CI) (*n*=1,824)
Number of Parental ACEs (Higher of Either Parent's Score)	1.25 (1.09, 1.43)**		1.22 (1.06, 1.39)**
Number of Parental PCEs(Higher of Either Parent's Score)		0.83 (0.72, 0.96)*	0.87 (0.75, 1.01)

*p-value < .05; **p-value < .01; ***p-value < .001

[a]Model adjusted for adult child age, sex, race, Latino ethnicity, highest level of parental education, highest level of parental income. A higher ACE score indicates more exposure to adverse childhood experiences. A higher PCE score indicates more exposure to positive childhood experiences.

ACE=adverse childhood experiences. PCE=positive childhood experiences. OR=odds ratio.

© 2023 Barnert ES et al. *JAMA Network Open.*
