# Supplementary material for: Parents’ Adverse and Positive Childhood Experiences and Offspring Involvement With the Criminal Legal System
Source: JAMA Netw Open. 2023 Oct 25;6(10):e2339648. doi: 10.1001/jamanetworkopen.2023.39648 (PMC10600584; doi:10.1001/jamanetworkopen.2023.39648)
Supplement: Supplement 2. — Data Sharing Statement [file jamanetwopen-e2339648-s002.pdf]

Data Sharing Statement

Barnert. Parents' Adverse and Positive Childhood Experiences and Offspring Involvement With the Criminal Legal System. *JAMA Netw Open*. Published October 25, 2023. doi:10.1001/jamanetworkopen.2023.39648

Data
Data available: No

Additional Information
Explanation for why data not available: PSID dataset is available through University of Michigan
